# Has endoscopic (TEP, TAPP) or open inguinal hernia repair a higher risk of bleeding in patients with coagulopathy or antithrombotic therapy? Data from the Herniamed Registry

**DOI:** 10.1007/s00464-015-4456-7

**Published:** 2015-08-15

**Authors:** F. Köckerling, C. Roessing, D. Adolf, C. Schug-Pass, D. Jacob

**Affiliations:** Department of Surgery and Center for Minimally Invasive Surgery, Academic Teaching Hospital of Charité Medical School, Vivantes Hospital, Neue Bergstraße 6, 13585 Berlin, Germany; StatConsult GmbH, Halberstädter Straße 40 a, 39112 Magdeburg, Germany

**Keywords:** Inguinal hernia repair, TEP, TAPP, Bleeding complication, Antithrombotic therapy, Coagulopathy

## Abstract

**Introduction:**

Inguinal hernia operations in the presence of antithrombotic therapy, based on antiplatelet or anticoagulant drugs, or existing coagulopathy are associated with a markedly higher risk for onset of postoperative secondary bleeding. To date, there is a paucity of concrete data on this important clinical aspect of inguinal hernia surgery. Up till now, the endoscopic (TEP, TAPP) techniques have been considered to be more risky because of the extensive dissection involved.

**Patients and methods:**

Out of the 82,911 patients featured in the Herniamed Hernia Registry who had undergone inguinal hernia repair, 9115 (11 %) were operated on while receiving antithrombotic therapy or with existing coagulopathy. The implications of that risk profile for onset of postoperative bleeding were investigated in multivariable analysis. In addition, other influence variables were identified.

**Results:**

The rate of postoperative secondary bleeding, at 3.91 %, was significantly higher in the risk group with coagulopathy or receiving antithrombotic therapy than in the group without that risk profile at 1.12 % (*p* < 0.001). Multivariable analysis revealed other influence variables which, in addition to coagulopathy or antithrombotic therapy, had a relevant influence on the occurrence of postoperative bleeding. These were open operation, a higher age, a higher ASA score, recurrence, male gender and a large hernia defect.

**Summary:**

Patients receiving antithrombotic therapy or with existing coagulopathy who undergo inguinal hernia operation have a fourfold higher risk for onset of postoperative secondary bleeding. Despite the extensive dissection required for endoscopic (TEP, TAPP) inguinal hernia repair, the risk of bleeding complications and complication-related reoperation appears to be lower.

Against a background of a progressively aging population, candidates for inguinal hernia repair are often elderly and have comorbidities. Therefore, it is not uncommon for the patients to be on antiplatelet or anticoagulant therapy [1]. Because antithrombotic agents are associated with longer bleeding time, the risk of postoperative hemorrhage is increased [1]. Prophylactic or therapeutic use of anticoagulants and platelet aggregation inhibitors confronts the treating surgeon with the challenge of protecting patients against thromboembolic complications without inducing bleeding complications. That calls for careful perioperative risk/benefit assessment with regard to the use of such therapeutics [2]. If it is possible to suspend platelet aggregation inhibitors for seven days or discontinue oral anticoagulant therapy and effect bridging with heparin, inguinal hernia surgery can be performed without increased risk of postoperative bleeding [3, 4]. But if, based on multidisciplinary consensus, antithrombotic medication cannot be dispensed with, a higher risk of bleeding complications must be countenanced. To date, there is a paucity of data on the risk associated with conduct of inguinal hernia operation in patients on antithrombotic therapy [5]. In addition to those patients receiving antithrombotic drug therapy, patients with coagulopathies, e.g., in the presence of cirrhosis of the liver, constitute a subgroup of individuals with a markedly higher risk profile for bleeding complications. The rate of bleeding complications secondary to inguinal hernia operations is given in the literature as being up to 7.9 % [6, 7]. In the Swedish Inguinal Hernia Registry, postoperative hematomas occurred in 3.5 % of 150,514 inguinal hernia operations [8]. Since endoscopic (TEP, TAPP) inguinal hernia repair needs extensive dissection at the preperitoneal space to place the mesh over the inguinal floor, there may be a higher risk of postoperative hemorrhage or hematoma compared with open repair [1].

Based on data from the Herniamed Registry [9], this present analysis now attempts to ascertain the rate of postoperative bleeding complications following inguinal hernia operations among patients with coagulopathy or receiving antiplatelet and anticoagulant therapy compared with that of patients who did not have a higher risk. Furthermore, it aims to identify other influence factors for the occurrence of bleeding complications in relation to inguinal hernia repair. It also endeavors to identify whether the risk of postoperative bleeding complications is really higher in the risk group with coagulopathy, anticoagulant or antiplatelet therapy following endoscopic (TEP, TAPP) repair compared with open operation.

## Patients and methods

Herniamed is a multicenter, internet-based Hernia Registry in which 383 participating clinics and surgeons in private practice from Germany, Austria and Switzerland (status: March 5, 2014) have prospectively registered their patients who had undergone hernia operation [9]. This present analysis now compares the prospective data of all patients who had undergone either unilateral or bilateral repair of an inguinal hernia between September 1, 2009, and March 5, 2014. Other inclusion criteria were a minimum age of 16 years, elective surgery and primary or recurrent operation. In total, 82,911 patients were enrolled. These comprised 69,508 patients with unilateral and 13,403 patients with bilateral inguinal repair. An open operation method was used in 35,370 of cases (Lichtenstein *n* = 22,926, Plug *n* = 3571, Shouldice *n* = 2919, Gilbert *n* = 2544, TIPP *n* = 1648, others *n* = 1762) and an endoscopic/laparoscopic technique in 47,541 cases (TAPP *n* = 29,292, TEP *n* = 18,249). The patient group at increased risk for onset of postoperative bleeding complications was defined as that comprising patients with either existing coagulopathy (e.g., in the presence of liver cirrhosis), currently receiving platelet aggregation inhibitor therapy or who had discontinued such therapy less than 7 days prior to the operation or patients whose Quick or INR value was not within the normal range during the operation due to treatment with coumarin. Since in a registry setting any increased risk for onset of postoperative bleeding complications can only be identified following inguinal hernia operation, no additional details, such as the product names of the platelet aggregation inhibitors, exact number of days they had been discontinued or the exact INR value, were recorded.

Other potential influence factors investigated were the surgical technique (endoscopic/open), age, sex, ASA status, hernia defect size based on EHS classification (grades I–III) and primary operation versus recurrence.

The outcome variable defined was postoperative secondary bleeding within 30 days of the operation. Postoperative secondary bleeding is defined as large surface bleeding into the skin surrounding the operation area and into the scrotum, hematomas in the operation area, major blood loss from indwelling drains and reoperations because of secondary bleeding. On one-year follow-up, patients are clearly asked again about the occurrence of such bleeding complications. In the case of bilateral inguinal hernias, postoperative secondary bleeding was deemed to have occurred if there was secondary bleeding on at least one of the two sides.

Unadjusted analysis was carried out to analyze an individual influence variable in respect of a target parameter. The main focus here was on the influence exerted by coagulopathy or antithrombotic therapy on increased bleeding risk. Fisher’s exact test was used for a categorical outcome variable, in particular since often individual number values were relatively small. However, it was not possible to use Fisher’s exact test for contingency tables of a higher dimension than 2 × 2 due to the large patient number. The asymptotic Chi-square test was used instead. The robust *t* test was used for continuous outcome variables that followed the normal distribution (Satterthwaite) to analyze the influence exerted by coagulopathy or antithrombotic therapy.

A binary logistic regression model was used to study the influence of patient (demographic) and surgery-related characteristics as well as of an increased bleeding risk associated with existing coagulopathy or antithrombotic therapy on the postoperative secondary bleeding rate, while the odds ratio with 95 % confidence interval was based on the Wald test. For influence variables with more than two categories, one of the latter forms was used in each case as reference category. For the continuous influence variable ‘age,’ the 10-year odds ratio is given.

## Results

Out of all patients who had undergone inguinal hernia operation, *n* = 9115/82,911 (11 %) had either existing coagulopathy or were still receiving effective antiplatelet or anticoagulant therapy. Out of these 9115 patients, *n* = 1207 (13.24 %) patients had coagulopathy, *n* = 1941 (21.29 %) a Quick or INR value that following coumarin therapy was outside the normal range, and *n* = 6641 (72.86 %) of patients had received treatment with platelet aggregation inhibitors which had been discontinued less than 7 days previously or had not at all been discontinued. As such, 33 out of 9115 patients had all three of these risk factors, 181 patients had received both coumarin derivatives and platelet aggregation inhibitors, 186 patients had existing coagulopathy in addition to receiving treatment with coumarin derivatives, and 241 patients had, in addition to existing coagulopathy, been treated with platelet aggregation inhibitors.

## Unadjusted analysis

Unadjusted analysis of the relationship of the risk group (coagulopathy, anticoagulant or antiplatelet therapy) and the non-risk group with the variables patient- and surgery-specific characteristics showed that there were highly significant differences between the risk group and the non-risk group with regard to all variables (in each case *p* < 0.001) (Table 1). For example, more open operations had been performed in the risk group (55.95 vs. 41.02 %). Besides, the proportion of male patients in the risk group was significantly greater (91.27 vs. 88.23 %). Likewise, there were significant differences in ASA classifications in that respect. In the risk group, the proportion of patients in ASA I was only 2.74 %, while in the non-risk group, it was 37.74 %. There were also significantly fewer cases of a small hernia defect (EHS I) in the risk group, at 10.61 %, compared with the non-risk group at 17.51 %. The proportion of recurrences in the risk group at 12.18 % was significantly higher than in the non-risk group at 10.55 % (Table 1). Furthermore, the proportion of bilateral operations in the risk group, at 12.59 %, was significantly lower than in the non-risk group, at 16.56 % (*p* < 0.001). Conversely, the use of drains in the risk group, at 37.03 %, was significantly higher than in the non-risk group, at 23.09 % (*p* < 0.001).

**Table 1:** Demographic and surgery-related parameters

		Coagulopathy, antithrombotic therapy				^*p*^
		Yes		No		
		*n*	%	*n*	%	
Operation	Laparoscopic	4015	44.05	43,526	58.98	<0.001
	Open	5100	55.95	30,270	41.02	
Sex	Male	8319	91.27	65,108	88.23	<0.001
	Female	796	8.73	8688	11.77	
ASA score	I	250	2.74	27,852	37.74	<0.001
	II	3911	42.91	38,318	51.92	
	III	4798	52.64	7470	10.12	
	IV	156	1.71	156	0.21	
Defect size	I (<1.5 cm)	967	10.61	12,920	17.51	<0.001
	II (1.5–3 cm)	4948	54.28	42,779	57.97	
	III (>3 cm)	3200	35.11	18,097	24.52	
Primary op	Yes	8005	87.82	66,012	89.45	<0.001
	No	1110	12.18	7784	10.55	
Bilateral	Yes	1180	12.95	12,223	16.56	<0.001
	No	7935	87.05	61,573	83.44	
EHS classification	Medial	2497	28.13	20,895	28.75	<0.001
	Lateral	4392	49.48	36,594	50.34	
	Femoral	111	1.25	1158	1.59	
	Combination	1877	21.14	14,043	19.32	
Drainage	Yes	3375	37.03	17,039	23.09	<0.001
	No	5740	62.97	56,757	76.91	

The patients in the risk group were on average 15 years older than in the non-risk control group (Table 2).

**Table 2:** Mean age and unadjusted *p*-value

		Coagulopathy, antithrombotic therapy		*p*
		Yes	No	
Age (years)	Mean ± SD	71.0 ± 10.6	55.6 ± 16.2	<0.001

The unadjusted test of the relationship between the presence of risk factors (coagulopathy, anticoagulant or antiplatelet therapy) and onset of postoperative secondary bleeding showed a significant difference of 3.91 versus 1.12 %; *p* < 0.001 to the disadvantage of the risk group (Table 3). The postoperative secondary bleeding rate for the entire patient collective was 1.42 %. Accordingly, the rate of complication-related reoperations in the risk group, at 2.26 %, was significantly higher than in the non-risk group, at 1.01 % (*p* < 0.001). The complication-related reoperation rate for the entire patient collective was 1.15 %.

**Table 3:** Postoperative bleeding and unadjusted *p* value

		Coagulopathy, antithrombotic therapy				Total		*p*
		Yes		No				
		*n*	%	*n*	%	*n*	%	
Postoperative bleeding	No	8759	96.09	72,971	98.88	81,730	98.58	<0.001
	Yes	356	3.91	825	1.12	1181	1.42	
Reoperation	No	8909	97.74	73,050	98.99	81,959	98.85	<0.001
	Yes	206	2.26	746	1.01	952	1.15	

## Multivariable analysis of postoperative bleeding in open and endoscopic inguinal hernia repair

The probability of postoperative secondary bleeding was determined primarily by the surgical technique used (*p* < 0.001) (Table 4). Conduct of an endoscopic operation resulted in significantly fewer cases of secondary bleeding (OR = 0.493 [0.431; 0.566]). A higher age increased the risk of postoperative secondary bleeding (10-year OR = 1.257 [1.196; 1.321], *p* < 0.001). Likewise, coagulopathy, anticoagulant or antiplatelet therapy had a highly significant impact on the risk of secondary bleeding (*p* < 0.001). The risk of postoperative secondary bleeding rose in the presence of these risk factors, with an odds ratio of OR = 2.001 [1.723; 2.323]. With an overall secondary bleeding rate of 1.4 %, that corresponds to the occurrence of postoperative secondary bleeding in around 19 out of 1000 patients with existing risk factors (coagulopathy, anticoagulant or antiplatelet therapy) compared with in 10 out of 1000 patients without that risk profile. Likewise, there were significantly more cases of secondary bleeding in the higher ASA categories (*p* < 0.001, e.g., ASA III vs. I: OR = 1.451 [1.187; 1.788]), as well as in male patients (OR = 1.244 [1.008; 1.536], *p* = 0.042). Finally, there was a tendency toward a higher secondary bleeding risk in the case of larger hernia defects.

**Table 4:** Multivariable analysis of postoperative bleeding in open and laparoscopic inguinal hernia repair

Parameter	*p*-value	Category	OR	[95 % CI]	
Operation	<0.001	Laparoscopic versus open	0.493	0.431	0.566
Coagulopathy, antithrombotic therapy	<0.001	Yes versus no	2.001	1.723	2.323
Age	<0.001		1.257	1.196	1.321
ASA score	<0.001	II versus I	0.966	0.815	1.146
		III versus I	1.451	1.178	1.788
		IV versus I	2.253	1.342	3.785
Primary-OP	<0.001	Yes versus no	0.749	0.632	0.888
EHS classification	0.001	Femoral versus combination	1.210	0.711	2.060
		Lateral versus combination	1.206	1.027	1.415
		Medial versus combination	0.907	0.759	1.085
Bilateral	0.005	Yes versus no	1.317	1.087	1.595
Sex	0.042	Male versus female	1.244	1.008	1.536
Defect size	0.121	I versus III	0.883	0.721	1.083
		II versus III	0.871	0.761	0.995

## Multivariable analysis of reoperations due to postoperative complications

The rate of complication-related reoperations was, first of all, negatively impacted by a bilateral operation (*p* < 0.001). Conduct of bilateral repair resulted in significantly more reoperations (OR = 2.168 [1.826; 2.574]) (Table 5). Likewise, a higher ASA classification (III vs. I: OR = 1.537 [1.224; 1.929]; IV vs. I: OR = 2.585 [1.365; 4.897]), existing coagulopathy, anticoagulant or antiplatelet therapy (OR = 1.561 [1.299; 1.874]) and higher age (10-year OR = 1.112 [1.055; 1.171]) led to a significantly higher risk of complication-related reoperation (*p* < 0.001). With a total reoperation rate of 1.15 %, this corresponds to a need for reoperation in around 14 out of 1000 patients in the risk group, and 9 out of 1000 patients in the non-risk group.

**Table 5:** Multivariable analysis of reoperations due to postoperative complications

Parameter	*p*-value	Category	OR	[95 % CI]	
Bilateral	<0.001	Yes versus no	2.168	1.826	2.574
ASA score	<0.001	II versus I	0.950	0.797	1.132
		III versus I	1.537	1.224	1.929
		IV versus I	2.585	1.365	4.897
Coagulopathy, antithrombotic therapy	<0.001	Yes versus no	1.561	1.299	1.874
Age	<0.001		1.112	1.055	1.171
Primary op	<0.001	Yes versus no	0.681	0.562	0.825
EHS classification	0.024	Femoral versus combination	1.686	1.032	2.754
		Lateral versus combination	1.134	0.952	1.351
		Medial versus combination	0.939	0.773	1.140
Defect size	0.024	I versus III	0.782	0.623	0.980
		II versus III	0.825	0.709	0.959
Operation	0.031	Laparoscopic versus open	0.848	0.730	0.985
Sex	0.460	Male versus female	0.922	0.743	1.144

Conduct of a primary operation (OR = 0.681 [0.562; 0.825]; *p* < 0.001, the presence of smaller hernia defects (*p* = 0.24; I vs. III: OR = 0.782 [0.623; 0.980]; II vs. III: OR = 0.825 [0.709; 0.959]) and a endoscopic operation (*p* = 0.031; OR = 0.848 [0.730; 0.985]) reduces the probability of complication-related reoperation.

## Multivariable analysis of postoperative bleeding in endoscopic inguinal hernia repair

There was a highly significant increase in the postoperative secondary bleeding risk associated with the 47,541 endoscopic surgical procedures in the presence of the risk factors coagulopathy, anticoagulant or antiplatelet therapy (OR = 2.110 [1.619; 2.749], *p* < 0.001) (Table 6). Hence, that corresponds to 13 cases of secondary bleeding for each 1000 endoscopic inguinal hernia operation in the risk group compared with six cases of secondary bleeding in patients without that risk profile, and it applies for an overall secondary bleeding rate of 0.9 % for endoscopic operations. A higher age (*p* < 0.001) as well as a higher ASA classification (*p* = 0.003) increased the secondary bleeding risk. The influence of gender as well as of recurrence was only tendentially reflected for the endoscopic data (*p* = 0.107 and *p* = 0.058, respectively), whereas the influence of hernia defect size was significantly revealed here (*p* = 0.015). A small hernia defect reduced secondary bleeding risk (e.g., I vs. III: OR = 0.646 [0.459; 0.910].

**Table 6:** Multivariable analysis of postoperative bleeding in laparoscopic inguinal hernia repair

Parameter	*p*-value	Category	OR	[95 % CI]	
Coagulopathy, antithrombotic therapy	<0.001	Yes versus no	2.110	1.619	2.749
Age	<0.001		1.192	1.102	1.290
EHS classification	<0.001	Femoral versus combination	1.459	0.647	3.293
		Lateral versus combination	1.204	0.932	1.555
		Medial versus combination	0.690	0.502	0.949
ASA score	0.005	II versus I	0.867	0.673	1.117
		III versus I	1.333	0.954	1.863
		IV versus I	2.617	0.789	8.685
Primary op	0.012	Yes versus no	0.689	0.515	0.923
Defect size	0.048	I versus III	0.682	0.480	0.967
		II versus III	0.787	0.629	0.986
Sex	0.062	Male versus female	1.438	0.982	2.106
Bilateral	0.137	Yes versus no	1.186	0.947	1.484

## Multivariable analysis of postoperative bleeding in open inguinal hernia repair

The strongest influence on onset of postoperative secondary bleeding in the 35,370 open operations was exerted by higher ASA classification, antithrombotic therapy or coagulopathy and bilateral operations (*p* < 0.001) (Table 7). Here, too, the presence of coagulopathy, anticoagulant or antiplatelet therapy significantly increased the risk of postoperative secondary bleeding (*p* < 0.001), with an odds ratio OR = 1.879 [1.576; 2.239]. With an overall secondary bleeding rate of around 2.1 % for open procedures, postoperative secondary bleeding thus occurs in 28 out of 1000 patients with the risk profile and in 13 out of 1000 patients without that profile. Likewise, the secondary bleeding rate rises in line with higher ASA classification and bilateral operation (*p* < 0.001). Here the influence of primary operation or recurrence and of gender can only be tendentially reflected (*p* = 0.062 and *p* = 0.113, respectively). The influence exerted by hernia defect size cannot be identified (*p* = 0.404).

**Table 7:** Multivariable analysis of postoperative bleeding in open inguinal hernia repair

Parameter	*p*-value	Category	OR	[95 % CI]	
Age	<0.001		1.301	1.220	1.387
Coagulopathy, antithrombotic therapy	<0.001	Yes versus no	1.940	1.619	2.324
ASA score	<0.001	II versus I	1.053	0.835	1.328
		III versus I	1.543	1.175	2.026
		IV versus I	2.252	1.253	4.046
Bilateral	0.001	Yes versus no	1.864	1.283	2.710
Primary op	0.024	Yes versus no	0.785	0.637	0.968
Sex	0.210	Male versus female	1.176	0.913	1.515
EHS classification	0.292	Femoral versus combination	1.064	0.526	2.154
		Lateral versus combination	1.178	0.959	1.449
		Medial versus combination	1.019	0.818	1.270
Defect size	0.463	I versus III	1.036	0.806	1.331
		II versus III	0.920	0.778	1.087

## Discussion

The present analysis investigated the influence exerted by coagulopathy or antithrombotic therapy on onset of postoperative secondary bleeding after inguinal hernia surgery. In doing so, it also identified other influence factors for the occurrence of secondary bleeding after inguinal hernia operation. To that effect, 82,911 patients who had undergone inguinal hernia operation and whose data were recorded in the Herniamed Registry were classified as belonging to either the risk group with existing coagulopathy/receiving antithrombotic therapy (11 %) or to a non-risk group (89 %), and were then compared with each other. Comparison revealed a significantly higher postoperative secondary bleeding rate of 3.91 % in the risk group versus a 1.12 % rate in the non-risk group with the total rate of 1.42 %. Other negative influence factors identified in multivariable analysis for onset of postoperative secondary bleeding were conduct of open inguinal hernia operation, higher patient age, higher ASA score, recurrence, male gender and a larger hernia defect. These results concord with an extent with those of the Swedish Hernia Registry. In the Swedish Hernia Registry, too, significantly more postoperative complications occurred in men, in patients with a higher age as well as in recurrences [8]. However, that study did not investigate the impact of different influence factors on individual postoperative complications. But an important difference between the registry data is that in the Swedish Hernia Registry more postoperative complications occurred after laparoscopic/endoscopic inguinal hernia operations than after open operations. In the Herniamed Registry, fewer cases of postoperative bleeding occurred after endoscopic procedures (TEP, TAPP) than after open operations. Hence, the complication-related reoperation rate following endoscopic inguinal hernia repair (TEP, TAPP) was significantly lower compared with the open operation. However, data analysis revealed that in the present study the proportion of endoscopic/laparoscopic inguinal hernia repairs was smaller in the risk group with coagulopathy or antithrombotic therapy (44.5 %) than in the non-risk group (58.98 %). Therefore, it must be assumed that an open surgical procedure was indicated more often in the risk group due to the presence of an elevated risk of bleeding complication. That means that the differential therapeutic approach taken in the risk group prior to surgery with regard to selecting either an open or a laparoscopic technique differed from that used in the non-risk group. It can thus be assumed that more patients with a poor risk profile for secondary bleeding were operated on using an open technique. That larger hernia defects in general entail more extensive dissection and accordingly result in a larger wound area, which helps to explain the influence exerted by the defect size on the probability of secondary bleeding. However, it was also noted that extensive dissection as required for endoscopic inguinal hernia repair does not necessarily lead to a higher rate of secondary bleeding and complication-related reoperations in patients with coagulopathy, anticoagulant or antiplatelet therapy compared with open surgery.

In summary, it can be noted that in the patient group undergoing inguinal hernia operation while receiving antithrombotic therapy or with existing coagulopathy, the risk for onset of postoperative secondary bleeding is fourfold higher than in patients without that risk profile. Other factors related to the individual patient and hernia type constitute an additional risk constellation for postoperative secondary bleeding. For example, a male patient with a high age, unfavorable ASA score, on antithrombotic therapy or with existing coagulopathy and a larger hernia defect or with recurrent hernia has the highest risk for onset of postoperative secondary bleeding. Accordingly, the choice of operation technique and of surgeon should be tailored to that risk profile. Conduct of inguinal hernia operations for patients on antithrombotic therapy or with existing coagulopathy requires a high level of attention and of expertise on the part of the surgeon. In this respect, the use of an endoscopic technique rather tends to reduce the risk of secondary bleeding and complication-related reoperations. The subtle dissection technique employed for the endoscopic repair procedure does appear to be associated with a lower risk of secondary bleeding compared with the open operation. Accordingly, when using subtle dissection and hemostasis techniques, endoscopic inguinal hernia repair can also be recommended for risk patients with coagulopathy, anticoagulant or antiplatelet therapy, and other risk factors (bilateral operations, recurrence, higher age, large hernia defects and male gender).

## Appendix: Herniamed Study Group

### Scientific board

**Köckerling**, Ferdinand (Chairman); **Berger**, Dieter; **Bittner**, Reinhard; **Fortelny**, René; **Koch**, Andreas; **Kraft,** Barbara; **Kuthe**, Andreas; **Lorenz**, Ralph; **Mayer**, Franz; **Moesta**, Kurt Thomas; **Niebuhr**, Henning; **Peiper**, Christian; **Pross**, Matthias; **Reinpold**, Wolfgang; **Simon**, Thomas; **Stechemesser**, Bernd; **Unger**, Solveig

### Participants

**Ahmetov**, Azat (Saint-Petersburg); **Alapatt,** Terence Francis (Frankfurt/Main); **Anders,** Stefan (Berlin); **Anderson**, Jürina (Würzburg); **Arndt**, Anatoli (Elmshorn); **Asperger,** Walter (Halle); **Avram**, Iulian (Saarbrücken); **Barkus**; Jörg (Velbert); **Becker**, Matthias (Freital); **Behrend**, Matthias (Deggendorf); **Beuleke,** Andrea (Burgwedel); **Berger,** Dieter (Baden–Baden); **Bittner,** Reinhard (Rottenburg); **Blumberg,** Claus (Lübeck); **Böckmann,** Ulrich (Papenburg); **Böhle**, Arnd Steffen (Bremen); **Böttger,** Thomas Carsten (Fürth); **Borchert,** Erika (Grevenbroich); **Born**, Henry (Leipzig); **Brabender,** Jan (Köln); **Breitenbuch von**, Philipp (Radebeul); **Brüggemann**, Armin (Kassel); **Brütting**, Alfred (Erlangen); **Budzier**, Eckhard (Meldorf); **Burghardt**, Jens (Rüdersdorf); **Carus**, Thomas (Bremen); **Cejnar**, Stephan-Alexander (München); **Chirikov**, Ruslan (Dorsten); **Comman,** Andreas (Bogen); **Crescent**i, Fabio (Verden/Aller); **Dapunt**, Emanuela (Bruneck); **Decker**, Georg (Berlin); **Demmel**, Michael (Arnsberg); **Descloux,** Alexandre (Baden); **Deusch**, Klaus-Peter (Wiesbaden); **Dick**, Marcus (Neumünster); **Dieterich**, Klaus (Ditzingen); **Dietz**, Harald (Landshut); **Dittmann**, Michael (Northeim); **Dornbusch**, Jan (Herzberg/Elster); **Drummer**, Bernhard (Forchheim); **Eckermann**, Oliver (Luckenwalde); **Eckhoff,** Jörn/Hamburg); **Elger**, Karlheinz (Germersheim); **Engelhardt**, Thomas (Erfurt); **Erichsen,** Axel (Friedrichshafen); **Eucker**, Dietmar (Bruderholz); **Fackeldey**, Volker (Kitzingen); **Farke**, Stefan (Delmenhorst); **Faust**, Hendrik (Emden); **Federmann**, Georg (Seehausen); **Feichter**, Albert (Wien); **Fiedler,** Michael (Eisenberg); **Fischer**, Ines (Wiener Neustadt); **Fortelny**, René H. (Wien); **Franczak**, Andreas (Wien); **Franke**, Claus (Düsseldorf); **Frankenberg von**, Moritz (Salem); **Frehner**, Wolfgang (Ottobeuren); **Friedhoff**, Klaus (Andernach); **Friedrich,** Jürgen (Essen); **Frings**, Wolfram (Bonn); **Fritsche**, Ralf (Darmstadt); **Frommhold,** Klaus (Coesfeld); **Frunder**, Albrecht (Tübingen); **Fuhrer**, Günther (Reutlingen); **Gassler,** Harald (Villach); **Gerdes**, Martin (Ostercappeln); **Gilg**, Kai-Uwe (Hartmannsdorf); **Glaubitz**, Martin (Neumünster); **Glutig,** Holger (Meißen); **Gmeiner**, Dietmar (Bad Dürrnberg); **Göring**, Herbert (München); **Grebe**, Werner (Rheda-Wiedenbrück); **Grothe**, Dirk (Melle); **Gürtler**, Thomas (Zürich); **Hache**, Helmer (Löbau); **Hämmerle**, Alexander (Bad Pyrmont); **Haffner**, Eugen (Hamm); **Hain**, Hans-Jürgen (Groß-Umstadt); **Hammans**, Sebastian (Lingen); **Hampe**, Carsten (Garbsen); **Harrer**, Petra (Starnberg); **Heinzmann**, Bernd (Magdeburg); **Heitland**, Tim (München); **Helbling**, Christian (Rapperswil); **Hempen**, Hans-Günther (Cloppenburg); **Henneking**, Klaus-Wilhelm (Bayreuth); **Hermes**, Wolfgang (Weyhe); **Herrgesell**, Holger (Berlin); **Herzing**, Holger Höchstadt); **Hessler**, Christian (Bingen); **Hildebrand**, Christiaan (Langenfeld); **Höferlin**, Andreas (Mainz); **Hoffmann**, Michael (Kassel; **Hofmann**, Eva M. (Frankfurt/Main); **Hopfe**r, Frank (Eggenfelden); **Hornung**, Frederic (Wolfratshausen); **Hügel**, Omar (Hannover); **Hüttemann**, Martin (Oberhausen); **Huhn**, Ulla (Berlin); **Imdahl**, Andreas (Heidenheim); **Jacob**, Dietmar (Bielefeld); **Jenert**, Burghard (Lichtenstein); **Jugenheimer**, Michael (Herrenberg); **Junger**, Marc (München); **Käs**, Stephan (Weiden); **Kahraman,** Orhan (Hamburg); **Kaiser,** Christian (Westerstede); **Kaiser**, Stefan (Kleinmachnow); **Kapischke**, Matthias (Hamburg); **Karch**, Matthias (Eichstätt); **Keck**, Heinrich (Wolfenbüttel); **Keller,** Hans W. (Bonn); **Kienzle**, Ulrich (Karlsruhe); **Kipfmüller**, Brigitte (Köthen); **Kirsch**, Ulrike (Oranienburg); **Klammer**, Frank (Ahlen); **Klatt**, Richard (Hagen); **Kleemann**, Nils (Perleberg); **Klein**, Karl-Hermann (Burbach); **Kleist**, Sven (Berlin); **Klobusicky**, Pavol (Bad Kissingen); **Kneifel**, Thomas (Datteln); **Knoop**, Michael (Frankfurt/Oder); **Knotter**, Bianca (Mannheim); **Koch,** Andreas (Cottbus); **Köckerling,** Ferdinand (Berlin); **Köhler**, Gernot (Linz); **König**, Oliver (Buchholz); **Kornblum**, Hans (Tübingen); **Krämer**, Dirk (Bad Zwischenahn); **Kraft**, Barbara (Stuttgart); **Kreissl**, Peter (Ebersberg); **Krones**, Carsten Johannes (Aachen); **Kruse,** Christinan (Aschaffenburg); **Kube**, Rainer (Cottbus); **Kühlberg**, Thomas (Berlin); **Kuhn,** Roger (Gifhorn); **Kusch,** Eduard (Gütersloh); **Kuthe,** Andreas (Hannover); **Ladberg**, Ralf (Bremen); **Ladra**, Jürgen (Düren); **Lahr-Eigen**, Rolf (Potsdam); **Lainka**, Martin (Wattenscheid); **Lammers**, Bernhard J. (Neuss); **Lancee**, Steffen (Alsfeld); **Larusson**, Hannes Jon (Pinneberg); **Lauschke**, Holger (Duisburg); **Leher,** Markus (Schärding); **Leidl**, Stefan (Waidhofen/Ybbs); **Lenz**, Stefan (Berlin); **Lesch**, Alexander (Kamp-Lintfort); **Lienert**, Mark (Duisburg); **Limberger**, Andreas (Schrobenhausen); **Locher**, Martin (Kiel); **Loghmanieh**, Siawasch (Viersen); **Lorenz**, Ralph (Berlin); **Mallmann**, Bernhard (Krefeld); **Manger**, Regina (Schwabmünchen); **Maurer**, Stephan (Münster); **Mayer**, Franz (Salzburg); **Menzel**, Ingo (Weimar); **Meurer**, Kirsten (Bochum); **Meyer**, Moritz (Ahaus**)**; **Mirow**, Lutz (Kirchberg); **Mittenzwey**, Hans-Joachim (Berlin); **Mörder-Köttgen**, Anja (Freiburg); **Moesta**, Kurt Thomas (Hannover); Moldenhauer, Ingolf (Braunschweig); **Morkramer**, Rolf (Xanten); **Mosa**, Tawfik (Merseburg); **Müller**, Hannes (Schlanders); **Münzberg**, Gregor (Berlin); **Mussack,** Thomas (St. Gallen); **Neumann**, Jürgen (Haan); **Niebuhr,** Henning (Hamburg); **Nölling**, Anke (Burbach); **Nostitz**, Friedrich Zoltán (Mühlhausen); **Obermaier**, Straubing); **Öz-Schmidt**, Meryem (Hanau); **Oldorf**, Peter (Usingen); **Olivieri**, Manuel (Pforzheim); **Pawelzik**, Marek (Hamburg); **Peiper**, Christian (Hamm); **Pertl**, Alexander (Spittal/Drau); **Philipp**, Mark (Rostock); **Pickart**, Lutz (Bad Langensalza); **Pizzera**, Christian (Graz); **Pöllath**, Martin (Sulzbach-Rosenberg); **Possin**, Ulrich (Laatzen); **Prenzel**, Klaus (Bad Neuenahr-Ahrweiler); **Pröve**, Florian (Goslar); **Pronnet**, Thomas (Fürstenfeldbruck); **Pross**, Matthias (Berlin); **Puff**, Johannes (Dinkelsbühl); **Rabl**, Anton (Passau); **Rapp**, Martin (Neunkirchen); **Reck**, Thomas (Püttlingen); **Reinpold,** Wolfgang (Hamburg); **Reuter**, Christoph (Quakenbrück); **Richter,** Jörg (Winnenden); **Riemann**, Kerstin (Alzenau-Wasserlos); **Rodehorst**, Anette (Otterndorf); **Roehr**, Thomas (Rödental); **Roncossek**, Bremerhaven); **Roth** Hartmut (Nürnberg); **Sardoschau**, Nihad (Saarbrücken); **Sauer**, Gottfried (Rüsselsheim); **Sauer**, Jörg (Arnsberg); **Seekamp**, Axel (Freiburg); **Seelig**, Matthias (Bad Soden); **Seiler**, Christoph Michael (Warendorf); **Seltmann,** Cornelia (Hachenburg); **Senkal,** Metin (Witten); **Shamiyeh**, Andreas (Linz); **Shang**, Edward (München); **Siemssen**, Björn (Berlin); **Sievers,** Dörte (Hamburg); **Silbernik**, Daniel (Bonn); **Simon**, Thomas (Sinsheim); **Sinn**, Daniel (Olpe); **Sinning**, Frank (Nürnberg); **Smaxwil**, Constatin Aurel (Stuttgart); **Schabel**, Volker (Kirchheim/Teck); **Schadd**, Peter (Euskirchen); **Schassen von**, Christian (Hamburg); **Schattenhofer**, Thomas (Vilshofen); **Scheidbach**, Hubert (Neustadt/Saale); **Schelp**, Lothar (Wuppertal); **Scherf**, Alexander (Pforzheim); **Scheyer**, Mathias (Bludenz); **Schimmelpenning,** Hendrik (Neustadt in Holstein); **Schinkel**, Svenja (Kempten); **Schmid**, Michael (Gera); **Schmid,** Thomas (Innsbruck); **Schmidt**, Rainer (Paderborn); **Schmidt**, Sven-Christian (Berlin); **Schmidt,** Ulf (Mechernich); **Schmitz**, Heiner (Jena); **Schmitz**, Ronald (Altenburg); **Schöche**, Jan (Borna); **Schoenen**, Detlef (Schwandorf); **Schrittwieser**, Rudolf/Bruck an der Mur); **Schroll**, Andreas (München); **Schultz**, Christian (Bremen-Lesum); **Schultz**, Harald (Landstuhl); **Schulze**, Frank P. Mülheim an der Ruhr); **Schumacher**, Franz-Josef (Oberhausen); **Schwab**, Robert (Koblenz); **Schwandner**, Thilo (Lich); **Schwarz**, Jochen Günter (Rottenburg); **Schymatzek**, Ulrich (Radevormwald); **Spangenberger**, Wolfgang (Bergisch-Gladbach); **Sperling**, Peter (Montabaur); **Staade**, Katja (Düsseldorf); **Staib**, Ludger (Esslingen); **Stamm**, Ingrid (Heppenheim); **Stark**, Wolfgang (Roth); **Stechemesser,** Bernd (Köln); **Steinhilper**, Uz (München); **Stern**, Oliver (Hamburg); **Stolte**, Thomas (Mannheim); **Stopinski,** Jürgen (Schwalmstadt); **Stubbe**, Hendrik (Güstrow/); **Stülzebach**, Carsten (Friedrichroda); **Tepel,** Jürgen (Osnabrück); **Terzić**, Alexander (Wildeshausen); **Teske,** Ulrich (Essen); **Thews**, Andreas (Schönebeck); **Tillenburg**, Wolfgang (Marktheidenfeld); **Timmermann,** Wolfgang (Hagen); **Train**, Stefan H. (Gronau); **Trauzettel**, Uwe (Plettenberg); **Triechelt**, Uwe (Langenhagen); **Ulcar**, Heimo (Schwarzach im Pongau); **Unger**, Solveig (Chemnitz); **Verweel**, Rainer (Hürth); **Vogel**, Ulrike (Berlin); **Voigt**, Rigo (Altenburg); **Voit**, Gerhard (Fürth); **Volkers**, Hans-Uwe (Norden); **Vossough**, Alexander (Neuss); **Wallasch**, Andreas (Menden); **Wallner**, Axel (Lüdinghausen); **Warscher,** Manfred (Lienz); **Warwas**, Markus (Bonn); **Weber**, Jörg (Köln); **Weiß**, Johannes (Schwetzingen); **Weißenbach**, Peter (Neunkirchen); **Werner**, Uwe (Lübbecke-Rahden); **Wessel,** Ina (Duisburg); **Weyhe**, Dirk (Oldenburg); **Wieber**, Isabell (Köln); **Wiesmann**, Aloys (Rheine); **Wiesner**, Ingo (Halle); **Woehe**, Fritz (Sanderhausen); **Wolf**, Claudio (Neuwied); **Yildirim**, Selcuk (Berlin); **Zarras**, Konstantinos (Düsseldorf); **Zeller**, Johannes (Waldshut-Tiengen); **Zhorzel**, Sven (Agatharied); **Zuz**, Gerhard (Leipzig);
